# Brentuximab vedotin compared with historical controls for severe skin involvement in cutaneous systemic sclerosis

**DOI:** 10.1093/rheumatology/keae460

**Published:** 2024-08-27

**Authors:** Andreu Fernández-Codina, Tatiana Nevskaya, Murray Baron, C Thomas Appleton, Matthew J Cecchini, Amanda Philip, Maha El-Shimy, Louise Vanderhoek, Iago Pinal-Fernández, Janet E Pope

**Affiliations:** Division of Rheumatology, Western University, London, ON, Canada; Division of General Internal Medicine-Windsor Campus, Western University, London, ON, Canada; Division of Rheumatology, Rheumatology Research Group, Vall d’Hebron University Hospital, Barcelona, Spain; Division of Rheumatology, Western University, London, ON, Canada; Division of Rheumatology, Jewish General Hospital, McGill University, Montreal, QC, Canada; Division of Rheumatology, Western University, London, ON, Canada; Division of Pathology, Western University, London, ON, Canada; Division of Rheumatology, Western University, London, ON, Canada; Division of Rheumatology, Western University, London, ON, Canada; Division of Rheumatology, Western University, London, ON, Canada; Muscle Unit, National Institute of Arthritis and Musculoskeletal and Skin Diseases, Bethesda, MD, USA; Department of Neurology, Johns Hopkins University School of Medicine, Baltimore, MD, USA; Division of Rheumatology, Western University, London, ON, Canada

Rheumatology key message

- Compared with historical controls, brentuximab vedotin may improve skin involvement and lung function in scleroderma.

Dear Editor, Treatment of severe skin involvement in patients with SSc is challenging. Studies have explored various immunosuppressants with a minimal effect on skin thickening [1]. Among treatments for dcSSc, autologous stem cell transplantation is the one showing the greatest improvement of the skin, leading to a significant decrease of the modified Rodnan skin score (mRSS) measurements. Nevertheless, there are risks and high costs associated with this treatment. We completed a phase II, open-label, single-arm trial in patients with dcSSc and severe skin disease, using brentuximab vedotin (an antibody-drug conjugate *vs* CD30 approved for non-Hodgkin’s lymphoma) [2]. In this *post hoc* analysis, we compared patients treated with brentuximab vedotin *vs* historic matched controls from the Canadian Scleroderma Research Group’s (CSRG) registry.

The investigator-initiated study was conducted in the Rheumatology division, Western University, London, ON, Canada and registered at ClinicalTrials.gov (NCT03198689). The drug supply and part of the funding were provided by Seagen, Inc. (now a wholly owned subsidiary of Pfizer, Inc.), who did not have any role in the design or interpretation of the results. Key inclusion criteria were: adults meeting the ACR/EULAR 2013 SSc classification criteria; mRSS ≥15; and active dcSSc, many of whom were previous immunosuppressive treatment failures. Individuals were treated with i.v. brentuximab vedotin 0.6 mg/kg every 3 weeks for 45 weeks, allowing background standard of care continuation. Age (±5 years), sex, mRSS and disease duration matched (±2 years) historic controls were obtained from the from the CSRG registry. Since there were a limited number of patients within the database with very high skin scores, matching controls was variable (1–3 controls per participant). We compared the data from a CSRG visit (week 0) matching a case from the brentuximab trial at week 0. Then the next yearly CSRG visit (week 52) was compared with study weeks 48 weeks. Mean and s.d., and frequency (%) were used to describe variables. To assess between groups differences, paired sample Student’s *t*-tests and independent *t*-tests or Fisher’s exact tests were performed.

Eleven cases were treated with brentuximab vedotin (nine completed the study). Their baseline characteristics have been reported [2]. Baseline characteristics of the brentuximab-treated patients and 26 matched controls are provided in Supplementary Tables S1–S3, available at *Rheumatology* online. Cases had been more exposed to prior immunosuppression (100 *vs* 54%) with higher patient general assessment scores of disease activity (6.2 *vs* 4.5, respectively). No significant differences were found between cases and controls regarding the use of standard of care immunosuppression during the study (73% *vs* 54%). The mRSS at baseline was numerically higher in cases (32 *vs* 27). At week 48/52 (cases/controls), mRSS was numerically lower for cases (21 *vs* 27), while ΔmRSS was statistically significantly better in the brentuximab patients [–11 *vs* –1, *P* < 0.001] (Fig. 1, Supplementary Table S4 and Fig. S1, available at *Rheumatology* online).

**Figure 1.**
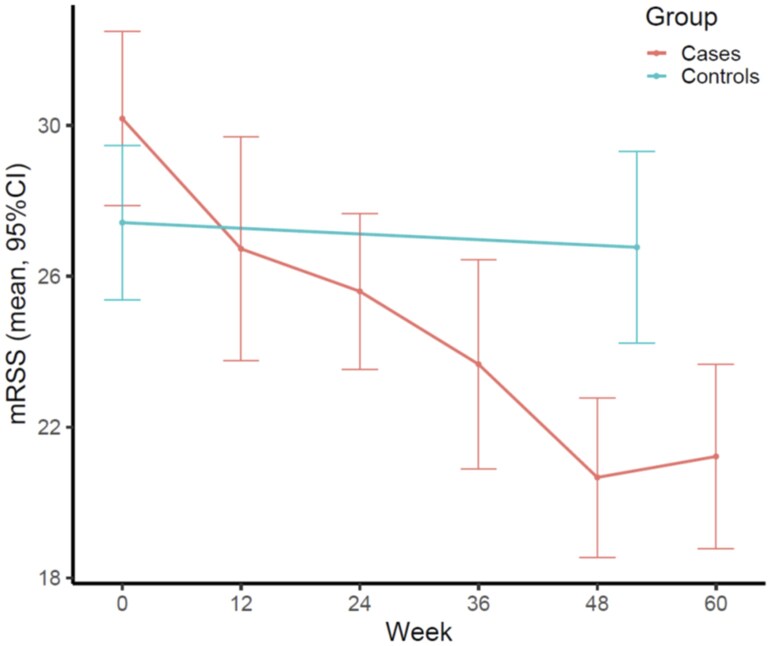
Evolution of the mean mRSS over time in patients treated with brentuximab vedotin and in historical matched controls from the Canadian Scleroderma Research Group (CSRG) database. mRSS: modified Rodnan skin score

The percentage forced vital capacity (FVC%) predicted trended to improve in the cases and worsened in controls (Supplementary Table S5, available at *Rheumatology* online); ΔFVC% (FVC% week 0 – FVC% week 48/52) statistically significantly improved in patients treated with brentuximab vedotin *vs* controls (8% *vs* –4%, *P* = 0.03) (Supplementary Fig. S2A, available at *Rheumatology* online).

Patient-reported outcomes interval change at week 48/52 improved significantly in cases (Supplementary Table S6, available at *Rheumatology* online). The Composite Response Index in diffuse cutaneous Systemic Sclerosis (CRISS) score at 48 weeks was 0.9 (0.4) for cases (*N* = 7) and 0.3 (0.4) for controls (*N* = 14) (*P* = 0.004) (Supplementary Fig. S2B, available at *Rheumatology* online).

Compared with controls, brentuximab vedotin showed a large improvement on the mRSS, and other benefits in lung function and patient-reported outcomes. These preliminary data suggest that patients with very high dcSSc often failing other immunosuppressive treatments could possibly benefit from brentuximab vedotin with respect to improving skin and lung function, and patient-reported outcomes [1, 3–5]. Favourable differences in achieving a CRISS score support the therapeutic effect of brentuximab vedotin.

This study has multiple limitations including the uncontrolled single-arm, unblinded open-label design. The CSRG matched controls are historic and may have been different. They had a lower prior exposure to immunosuppression compared with the brentuximab patients. Furthermore, the study was underpowered to assess for differences in mRSS based on previous or concomitant treatment. It was difficult to get matched controls given the very high mRSS of the brentuximab patients.

In conclusion, brentuximab vedotin improved the mRSS in patients with severe dcSSc. In this exploratory analysis comparing the results to matched historic controls, we observed skin, lung and patient-reported outcomes improvements relative to controls receiving standard of care. These data could suggest that a well-designed randomized double-blinded trial should be considered, as safety and tolerability were also demonstrated [2] in dcSSc patients who received brentuximab vedotin.

## Supplementary Material

keae460_Supplementary_Data

## Data Availability

Supporting information is available in the Supplementary Appendix, available at *Rheumatology* online, and further data are available at any time from the corresponding author on request including de-identified patient data.
