# Supplementary material for: Brentuximab vedotin compared with historical controls for severe skin involvement in cutaneous systemic sclerosis
Source: Rheumatology (Oxford). 2024 Aug 27;64(2):888–9. doi: 10.1093/rheumatology/keae460 (PMC11781570; doi:10.1093/rheumatology/keae460)

Inclusion criteria:

1. Patients with scleroderma, aged 18 years or older.
2. Subjects met the 2013 ACR/EULAR classification criteria.
3. Early dcSSc (within 5 years of first non-RP symptom) or active dcSSc as determined by worsening mRSS, presence of tendon friction rubs, and/or elevated inflammatory markers thought to be due to active dcSSc and not related to other issues such as infection.
4. mRSS ≥15.
5. Able to give informed consent.
6. Negative TB skin test at screening, or treatment with isoniazid for 6 months or other standardized latent tuberculosis (TB) treatment in the past.

Exclusion Criteria:

1. Poor pulmonary function (FVC <40% and/or DLCO <30%).
2. Pregnancy, breast feeding, or childbearing potential without practicing reliable contraception (and partners for men in the study).
3. Clinically significant pulmonary hypertension requiring drug therapy.
4. Clinically significant cardiac disease.
5. Chronic or ongoing active infectious disease requiring systemic treatment including
   1. Seropositivity for human immunodeficiency virus at study entry.
   2. Active TB infection.
   3. Active viral infection with viral replication of hepatitis B or C virus at study entry.
6. Significant concurrent, uncontrolled medical condition including, but not limited to renal, hepatic, pancreatic, haematological, gastrointestinal, endocrine, pulmonary, neurological, cerebral or psychiatric disease, and/or cancer.
7. Peripheral neuropathy at screening Grade 2 or higher.
8. Patients known or suspected of not being able to comply with a study protocol (e.g. due to alcoholism, drug dependency, or severe psychological disorder)
9. Any of the following laboratory abnormalities at screening:
   -Absolute neutrophils count <2.0 x 109/L
   -Hemoglobin <85 g/L
   -Platelet count <100 x 109/L
   -AST/SGOT >2-fold upper normal level
   -ALT/SGPT >2-fold upper normal level
10. Participation in another clinical trial within six weeks before randomization in this study
11. Use of rituximab within the previous 4 months.
12. Immunization with a live/attenuated vaccine less than 4 weeks prior to the baseline visit.
13. Previous use of brentuximab vedotin.
14. Current or history of progressive multifocal leukoencephalopathy.

Supplementary Table 1. Individualized description and evolution of controls.

| **Ctrl** | **Age (yrs)** | **Gender** | **DD (yrs)** | **Past IS** | **Current IS** | **mRSS week 0** | **mRSS week 48** | **ΔmRSS week 48** |
| --- | --- | --- | --- | --- | --- | --- | --- | --- |
| **1** | 73 | F | 4.8 | MTX | MTX | 25 | 14 | -11 |
| **2** | 65 | F | 6.5 | GC, MTX | MTX | 27 | 40 | -13 |
| **3** | 67 | F | 6.7 | - | - | 27 | 15 | -12 |
| **4** | 69 | F | 4.8 | GC | GC | 35 | 25 | -10 |
| **5** | 56 | F | 4.4 | MTX | - | 37 | 29 | -8 |
| **6** | 52 | F | 4.2 | CYC | CYC | 35 | 34 | -1 |
| **7** | 57 | F | 5.7 | GC | GC | 25 | 29 | 4 |
| **8** | 55 | F | 4.6 | GC, MTX | GC, MTX | 29 | 48 | 19 |
| **9** | 56 | F | 6.4 | - | - | 24 | 27 | 3 |
| **10** | 57 | F | 8.8 | MTX | MTX | 25 | 26 | 1 |
| **11** | 54 | F | 5.3 | GC, TNF | GC, TNF | 25 | 35 | 10 |
| **12** | 38 | M | 3.1 | - | - | 37 | 22 | -15 |
| **13** | 61 | M | 1.1 | GC, MTX | GC, MTX | 42 | 34 | -8 |
| **14** | 64 | M | 1.5 | GC | GC | 33 | 30 | -3 |
| **15** | 59 | F | 8.1 | - | - | 19 | 13 | -6 |
| **16** | 64 | F | 9 | - | - | 17 | 16 | -1 |
| **17** | 63 | F | 7.6 | - | - | 17 | 17 | 0 |
| **18** | 32 | F | 11.5 | MMF | MMF | 21 | 19 | -2 |
| **19** | 35 | F | 11.5 | - | MTX | 20 | 17 | -3 |
| **20** | 45 | F | 4 | CYC, AZA | CYC | 23 | 31 | 8 |
| **21** | 45 | F | 2.1 | - | - | 22 | 25 | 3 |
| **22** | 37 | F | 2.1 | - | - | 18 | 24 | 6 |
| **23** | 50 | F | 1.8 | - | - | 38 | 41 | 3 |
| **24** | 60 | F | 1.6 | MTX | CYC, AZA, MTX | 35 | 36 | 1 |
| **25** | 67 | M | 1.4 | - | - | 35 | 31 | -4 |
| **26** | 65 | M | 1.8 | - | - | 22 | 18 | -4 |

Ctrl=control; yrs=years; DD=disease duration; F=female; M=male; IS=immunosuppression; mRSS=modified Rodnan skin score; MTX=methotrexate; GC=glucocorticoids; CYC=cyclophosphamide; TNF=tumor necrosis factor inhibitor; MMF=mycophenolate mofetil; AZA=azathioprine

Supplementary Table 2. Differences in the use of immunosuppression before and during the study period among cases and controls

| **Immunosuppressor** | **Past immunosuppression** | | | **Current immunosuppression** | | |
| --- | --- | --- | --- | --- | --- | --- |
|  | **Cases**  **N=11**  **(%)** | **Controls**  **N=26**  **(%)** | **P-value** | **Cases**  **N=11**  **(%)** | **Controls**  **N=26**  **(%)** | **P-value** |
| **Glucocorticoids** | 3 (27) | 7 (27) | 1 | 0 (0) | 7 (27) | 0.08 |
| **Cyclophosphamide** | 4 (36) | 2 (8) | 0.05 | 0 (0) | 3 (12) | 0.5 |
| **Methotrexate** | 11 (100) | 7 (27) | 0.001 | 4 (36) | 7 (27) | 0.7 |
| **Azathioprine** | 4 (36) | 1 (4) | 0.02 | 1 (9) | 1 (4) | 0.5 |
| **Mycophenolate mofetil** | 7 (64) | 1 (4) | 0.001 | 4 (36) | 1 (4) | 0.02 |
| **Rituximab** | 1 (9) | 0 (0) | 0.3 | 0 (0) | 0 (0) | 1 |
| **Abatacept** | 1 (9) | 0 (0) | 0.3 | 0 (0) | 0 (0) | 1 |
| **TNF inhibitor** | 0 (0) | 1 (4) | 1 | 0 (0) | 1 (4) | 1 |
| **Nintedanib** | 0 (0) | 0 (0) | 1 | 1 (9) | 0 (0) | 0.3 |
| **Any immunosuppression** | 11 | 14 | 0.007 | 8 | 14 | 0.5 |

N=number of patients

Supplementary Table 3. Baseline characteristics of systemic sclerosis patients treated with brentuximab (cases) and historical controls

|  | **Cases N=11** | **Controls n=26** | **P-value** |
| --- | --- | --- | --- |
| Women N (%) | 8 (72.7) | 21 (80.8) | 0.7 |
| Previous immunosuppressive treatment | 11 (100) | 14 (53.8) | 0.007 |
| Interstitial lung disease | 5 (45.5) | 11 (42.3) | 0.9 |
| Age in years  Mean (SD) | 58.6 (13.5) | 55.6 (11.1) | 0.5 |
| Disease duration in years | 4.7 (3.4) | 5 (3.1) | 0.8 |
| mRSS | 30.2 (8.3) | 27.4 (7.4) | 0.3 |
| FVC% | 79.1 (26.6) | 88.8 (16.8) | 0.2 |
| PGA | 6.2 (2.5) | 4.5 (2.2) | 0.048 |
| MDGA severity | 5 (2.1) | 4.1 (2) | 0.2 |
| HAQ-DI | 1.455 (0.799) | 1.479 (0.483) | 0.9 |

N=number of patients; SD=standard deviation; mRSS=modified Rodnan skin score; FVC%=forced vital capacity % N=11 cases and 20 controls; CRP=c reactive protein; PGA=patient global assessment; MDGA=medical doctor global assessment; HAQ-DI=health assessment questionnaire-disability index N=11 cases and 24 controls; FACIT=functional assessment of chronic illness therapy-fatigue

Supplementary Table 4. Modified Rodnan skin score evolution for patients treated with brentuximab vedotin and controls. Per protocol analysis

| **mRSS Mean (SD)** | **Time 1** | **Time 2** | **Mean difference** |
| --- | --- | --- | --- |
| Cases week 0-12 | 30.2 (7.9) | 28.2 (10.4) | 3.8 (95% CI 1.1, 6.4), P=0.015 |
| Cases week 0-24 | 30.2 (7.9) | 25.6 (7.9) | 6.4 (95% CI 3, 9.9), P=0.002 |
| Cases week 0-36 | 30.2 (7.9) | 23.7 (10) | 8.3 (95% CI 3.9, 13) P=0.002 |
| Cases week 0-48 | 30.2 (7.9) | 20.7 (7.6) | 11 (95% CI 6.9, 16), P<0.001 |
| Cases week 0-60 | 30.2 (7.9) | 21.2 (8.8) | 11 (95% CI 5.3, 16), P=0.002 |
| Cases week 48-60 | 20.7 (7.6) | 21.2 (8.8) | -0.6 (95% CI -2.5, 1.4), P=0.5 |
| Controls week 0-48/52 | 27.4 (7.4) | 26.8 (9.2) | 0.6 (95% CI -2.6, 3.9), P=0.7 |
|  | **Cases**  **N=9** | **Controls**  **N=26** | **P-value** |
| Week 0 | 32 (7.9) | 27.4 (7.4) | 0.12 |
| Week 48 | 20.7 (7.6) | 26.8 (9.2) | 0.084 |
| ΔmRSS week 0-48/52 | -11.3 (5.8) | -0.7 (7.9) | <0.001 |

mRSS=modified Rodnan skin score; SD=standard deviation; CI=confidence interval. No data was available for controls at week 12, 24, 36 or 48

Supplementary Figure 1. Individualized evolution of mRSS during the study for cases and controls


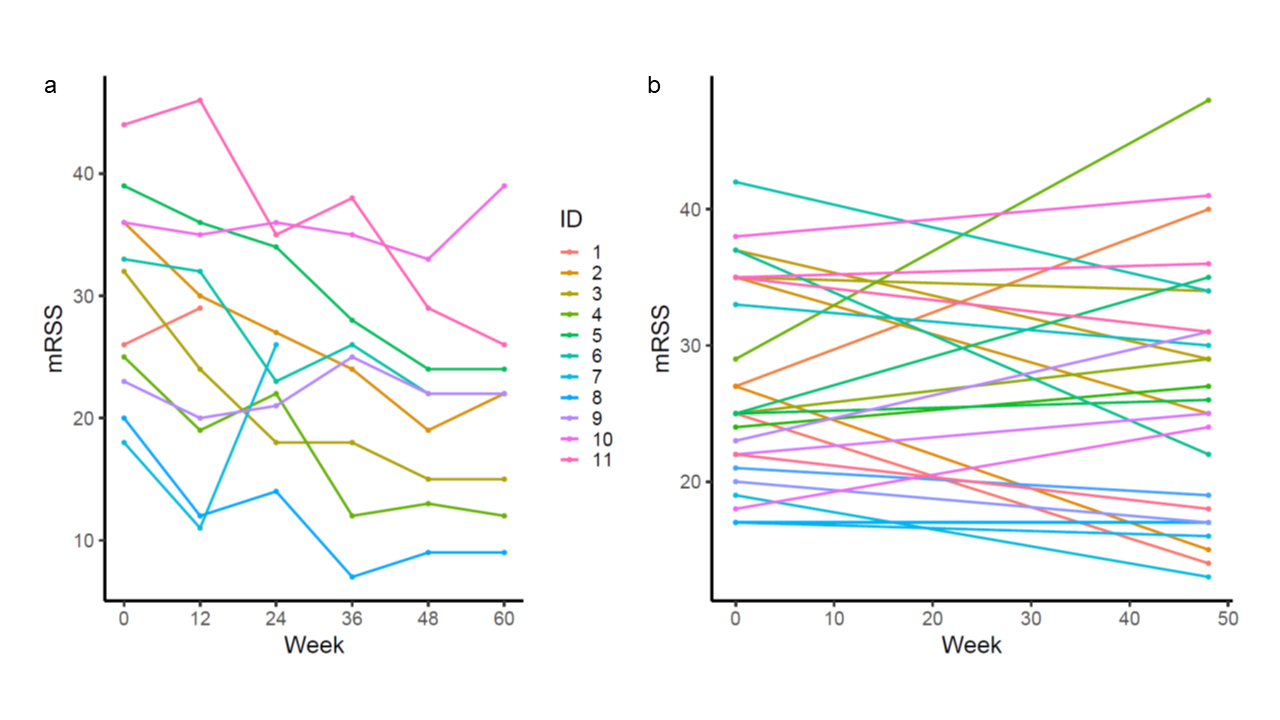


Panel a shows the change in mRSS in patients treated with brentuximab vedotin between week 0 and week 60. Panel b shows the change in mRSS in controls between baseline (week 0) and week 52. mRSS=modified Rodnan skin score; ID=identification

Supplementary Table 5. Pulmonary function tests in patients treated with brentuximab vedotin and controls

| **Cases  N=9**  **Mean (SD)** | **Before week 0** | **After week 48** | **Mean difference** |
| --- | --- | --- | --- |
| FVC% | 80.8 (23.8) | 88.6 (26.2) | -7.8 (95% CI -17, 1.9); P=0.1 |
| **Controls N=17** | **Week 0** | **Week 52** | **Mean difference** |
| FVC% | 88 (16.8) | 86 (18.9) | 3.8 (95% CI -1.6, 9.2); P=0.2 |
|  | **Cases** | **Controls** | **Mean difference** |
| ΔFVC% | 7.8 (12.5) | -4 (11.5) | 12 (95% CI 1.1, 22.9); P=0.03 |

N=number; SD=standard deviation; FVC=forced vital capacity; ΔFVC%=difference in forced vital capacity %; 95% CI=95% confidence interval

PFTs for cases were performed a mean of 28.7 (SD 21) weeks before the study onset and at 55.5 (SD 14) weeks after the study start date.

Supplementary Table 6. Patient/physician reported outcomes for patients treated with brentuximab vedotin and controls

| **Cases Mean (SD)** | **Week 0** | **Week 48** | **Mean difference** |
| --- | --- | --- | --- |
| PGA N=8 | 7 (2) | 4.4 (2.7) | 2.4 (95% CI 0.8, 4); P=0.008 |
| MDGA severity N=8 | 5.6 (1.9) | 2.1 (1.1) | 2.7 (95% CI 1.1, 4.4); P=0.007 |
| HAQ-DI N=8 | 1.708 (0.612) | 1.422 (0.858) | -0.31 (95% CI -0.14, 0.76); P=0.14 |
| **Controls** | **Week 0** | **Week 48** | **Mean difference** |
| PGA N=26 | 4.5 (2.2) | 4.5 (2.7) | 0.04 (95% CI -0.8, 0.4); P>0.9 |
| MDGA severity N=26 | 4.1 (2) | 4.2 (2.5) | -0.24 (95% CI -1.2, 0.7); P=0.078 |
| HAQ-DI N=26 | 1.748 (0.483) | 1.568 (0.55) | -0.09 (95% CI -0.3, 0.07); P>0.9 |
|  | **Cases** | **Controls** | **P-value** |
| ΔPGA | -2.4 (1.9) | -0.04 (2.1) | 0.008 |
| ΔMDGA severity | -2.7 (1.8) | 0.2 (2.4) | 0.005 |
| ΔHAQ-DI | -0.313 (0.539) | 0.089 (0.384) | 0.028 |
|  |  |  |  |

SD=standard deviation; PGA=patient general assessment (0-10); MDGA=physician general assessment (0-10); HAQ-DI=health assessment questionnaire-disability index (0-3); CI=confidence interval

Supplementary Figure 2. Differences in forced vital capacity and composite response index in diffuse cutaneous systemic sclerosis between patients treated with brentuximab vedotin and controls

Panel a depicts the difference in forced vital capacity percentage (Difference of FVC%) between patients treated with brentuximab vedotin and controls at the end of the study (weeks 48/52). Panel b shows the comparison of the composite response index in diffuse cutaneous systemic sclerosis (CRISS) in patients treated with brentuximab vedotin (48 weeks) versus controls (52 weeks)


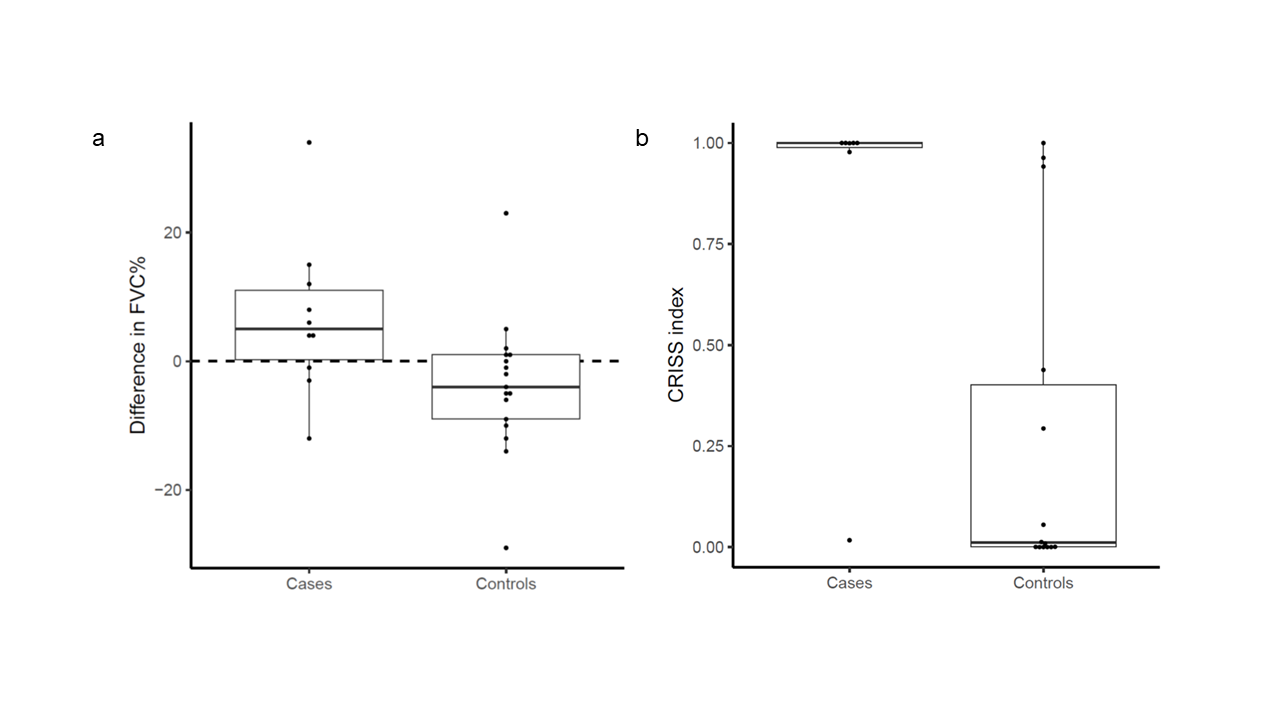

Supplement: keae460_Supplementary_Data [file keae460_supplementary_data.zip › rhe-24-1613-File005.docx]
