# Supplementary figures and images for: Brentuximab vedotin compared with historical controls for severe skin involvement in cutaneous systemic sclerosis
Source: Rheumatology (Oxford). 2024 Aug 27;64(2):888–9. doi: 10.1093/rheumatology/keae460 (PMC11781570; doi:10.1093/rheumatology/keae460)

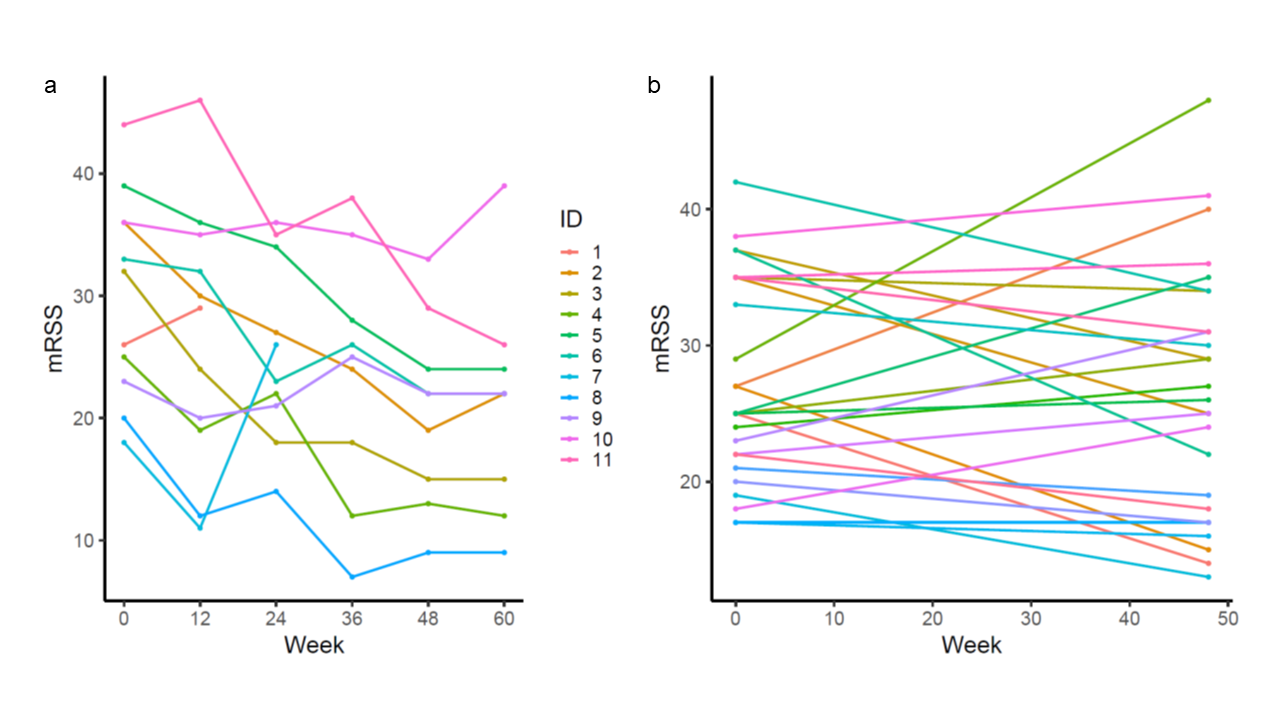

Supplement: keae460_Supplementary_Data [file keae460_supplementary_data.zip › rhe-24-1613-File003.tif]

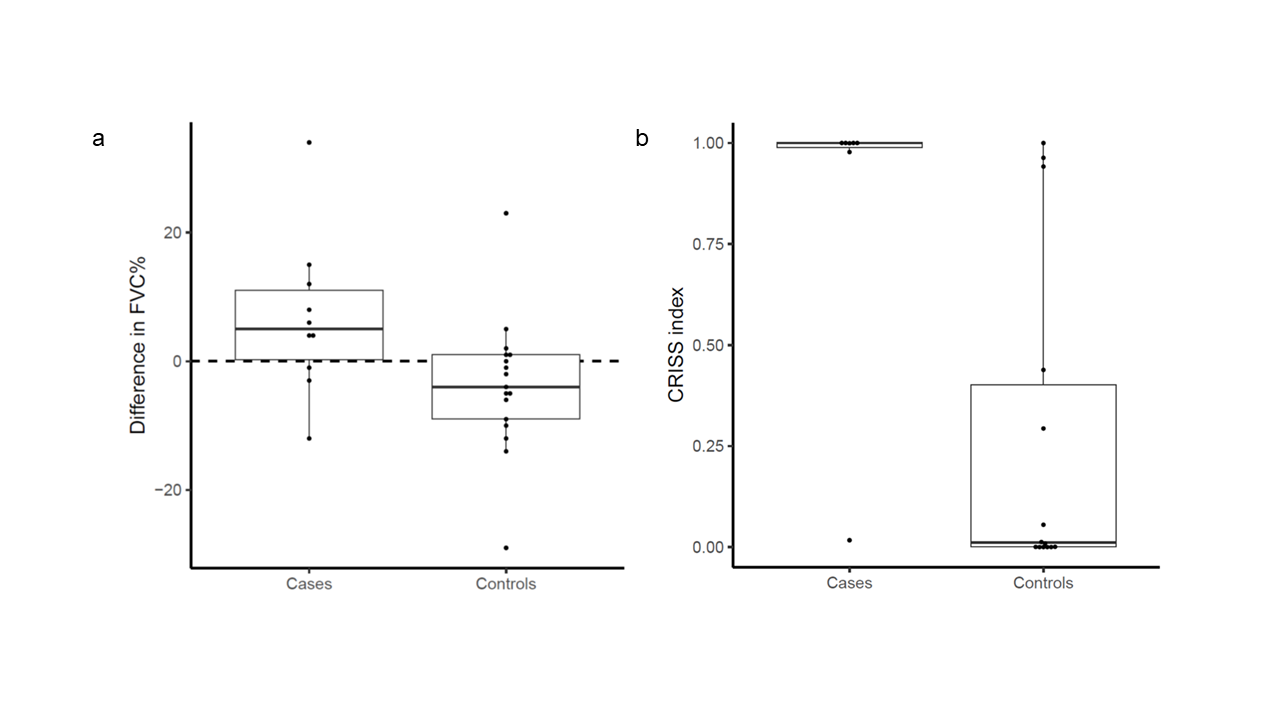

Supplement: keae460_Supplementary_Data [file keae460_supplementary_data.zip › rhe-24-1613-File004.tif]
